# Assessment of Burden on Family Caregivers of Children With Sickle Cell Anemia in Al Madinah Al Munawwarah, Saudi Arabia

**DOI:** 10.7759/cureus.66160

**Published:** 2024-08-05

**Authors:** Yousef Aloufi, Sami Al-Dubai, Asim A Alamri, Abdulghani Lodhi, Saeed S Alammari, Fayez Aloufi

**Affiliations:** 1 Preventive Medicine, Al Madinah Health Cluster, Ministry of Health, Madinah, SAU; 2 Preventive Medicine Post Graduate Studies, Al Madinah Health Cluster, Ministry of Health, Madinah, SAU; 3 Pediatric Hematology and Oncology, King Salman Medical City, Madinah, SAU; 4 Neonatology, Ministry of National Guard, Madinah, SAU; 5 Preventive Medicine, Al Madinah Health Cluster, Ministry of Health, Al Madinah, SAU; 6 Endodontics, King Fahad General Hospital, Specialized Dental Center, Madinah Health Cluster, Madinah, SAU

**Keywords:** sickle cell anemia, quality of life, family, children, caregiver burden

## Abstract

Background

Sickle cell anemia (SCA) results in various complications, necessitating continuous daily care and placing burdens on caregivers.

Objectives

This study aims to assess the burden on family caregivers of children with SCA and its associated factors.

Materials and methods

This analytical cross-sectional study was conducted in Madinah City, Saudi Arabia. We included family caregivers of children with SCA who were registered and treated at the Maternity and Child Hospital in King Salman Medical City. Data were collected from all registered files of children who received treatment for SCA. Data from participants was obtained using the validated Arabic version of the Zarit Burden Interview (ZBI). Descriptive statistics, chi-square tests, independent sample t-tests, and multivariate regression analysis were used in the statistical analysis.

Results

Overall, 124 caregivers participated out of 166 (response rate: 74.7%), among which 83 (66.9%) were fathers, 72 (58.1%) were aged ≥40 years, 96 (77.4%) held Saudi nationality, and 62 (50%) had a monthly income of <5000 SAR. The average daily caregiving hours were 5±4 hours, and 30 (24.2%) of children were diagnosed with associated physical or psychological diseases. The Zarit Burden Interview score indicated that 45 (36.3%) of caregivers reported no burden, whereas 51 (41.1%), 22 (17.7%), and 6 (4.8%) reported mild, moderate, and severe burden, respectively. Factors contributing to the burden included being a mother, low financial resources, non-Saudi nationality, children diagnosed with associated physical or psychological diseases, and caregiving hours.

Conclusions

The burden on SCA caregivers was higher for caregivers who were mothers, non-Saudis, those with lower income, and children with physical or psychological diseases, as well as more caregiving hours. Enhancing the overall well-being of families affected by the SCA burden involves creating targeted interventions and comprehensive support programs.

## Introduction

Sickle cell anemia (SCA) is an inherited chronic hemoglobinopathy that results in various complications, necessitating continuous daily care [1]. Approximately 300,000 children are born with SCA worldwide [2]. Regional variations in prevalence exist in Saudi Arabia [3]. Caring for children with SCA introduces difficulties for caregivers [4], with many exhibiting psychological distress [5,6]. A previous study revealed that 54% of caregivers were worried about worsening symptoms [7]. Various caregiving responsibilities for children with SCA, such as administering medications, providing assistance during disease complications and crises, transporting the children to clinics and hospitals for treatment and visits, as well as managing communication with healthcare providers and schools, contribute to caregiver burden [5,8,9].

Caregiver burden refers to the negative impact and strain experienced by individuals who provide care to a family member with a chronic illness or disability, such as SCA. It encompasses physical, emotional, social, and financial challenges that caregivers face in fulfilling their roles, manifesting as stress, exhaustion, frustration, sadness, and being overwhelmed, which affect social relationships, occupational functioning, and the personal wellbeing of caregivers [8,9]. Factors such as being a mother, low financial resources, frequent painful crises, and a lack of social assistance have been reported to influence caregiver burden [8,9].

Understanding the caregiver burden and identifying its contributing factors are crucial for improving the well-being of this population and may aid in developing policies, promoting evidence-based knowledge regarding the disease burden in the community, and encouraging premarital screening. To the best of our knowledge, no previous studies have explored the burden of caregivers of children with SCA in Madinah City, Saudi Arabia. Therefore, in this study, we aimed to assess this burden and its associated factors at the Maternity and Child Hospital in King Salman Medical City, Al Madinah Al Munawwarah, Saudi Arabia.

## Materials and methods

Study setting and population

This was an analytical cross-sectional study conducted from December 2023 to June 2024 at the Maternity and Child Hospital in King Salman Medical City, Al Madinah Al Munawwarah, Saudi Arabia. Caregivers of children with SCA were identified using the hospital’s database. All children with SCA registered in the hospital were recruited for this study, totaling 166 patients. Among these patients, 40 did not respond, and two refused to participate.

Inclusion and exclusion criteria

Children who were registered and received treatment at the hospital, aged ≤14 years, and resided in Al Madinah Al Munawwarah City were eligible for participation. Eligible caregivers included those who were providing care to children with SCA registered and treated at the same hospital, aged ≥18 years, and resided in Al Madinah Al Munawwarah City. Children in governmental social institutions (orphanages) were excluded. Caregivers who had been caring for affected children for <1 year and those who did not understand Arabic or English were also excluded.

Ethical considerations

This study was approved by the Ethics Committee of King Salman Medical City (approval number IRB23-055). Informed consent was obtained from the caregivers of each child before participation. We informed the participants that their involvement was voluntary, they had the right to withdraw from the study at any time without negative consequences, and we would treat all the information they provided in the questionnaire confidentially. Furthermore, it was emphasized that participation in the study had no potential harm.

Data collection

The data collection method involved contacting eligible participants via phone calls to obtain permission to participate in the study and ensure they understood the purpose of the study. Upon confirmation of their participation, a clear message was sent through WhatsApp, along with a Google Forms link containing the questionnaire and consent form, requesting that they complete it. Participants who did not respond initially were sent follow-up phone calls and messages as reminders to ensure participation.

Study tool

A self-administered questionnaire comprising three parts was used as the study tool. The first and second parts included the sociodemographic data of caregivers and children, respectively, whereas the third part included the validated Arabic version of the Zarit Burden Interview (ZBI) for the assessment of caregiver burden. The ZBI questionnaire was distributed in Arabic and constituted 22 questions, each rated on a 5-point Likert-type scale (0 [never] to 4 [nearly always]; total score range: 0-88). The level of burden was classified according to the total score as follows: 0-20, little or no burden; 21-40, mild to moderate burden; 41-60, moderate to severe burden; and 61-88, severe burden [10].

The ZBI was initially developed in 1980 to evaluate the burden experienced by the caregivers of patients with Alzheimer’s disease. The ZBI has since become a widely used tool for evaluating caregiver burden and has been adapted into multiple languages. It has been extensively applied in clinical studies with published reports on caregivers of individuals with various physical and mental conditions, including SCA [10,11]. The psychometric properties of the ZBI have been extensively evaluated in caregivers, and the validity parameters for the Arabic version of the ZBI (Cronbach’s alpha = 0.8) and Arabic translations of the ZBI have good internal consistency and reliability [12]. In this study, caregiver burden was defined as a ZBI score of >41.

Statistical analysis

All statistical analyses were performed using SPSS Statistics (version 29). The distribution of participants’ responses to questionnaire items was reported using descriptive statistics. Continuous data are presented as means and standard deviations, whereas categorical data are expressed as frequencies and percentages. The chi-squared and independent sample t-tests were used for inferential statistics. A multivariate logistic regression analysis was conducted. A 95% confidence interval (CI) was calculated, and statistical significance was set at a p-value of <0.05. The level of burden was assessed using the ZBI (the dependent variable) and categorized into two groups: no burden (scores of 0 to 40) and burden (scores of 41 to 88).

## Results

Of 166 caregivers, 124 participated in the study (response rate, 74.7%). Overall, 83 (66.9%) of the caregivers were fathers, 72 (58.1%) were aged >40 years, 96 (77.4%) held a Saudi nationality, and 115 (92.7%) were married. Among all respondents, 52 (41.9%) had a university degree or higher, 63 (50.8%) were unemployed, and 62 (50%) had a monthly income of <5000 SAR (Table 1). The average number of family members, daily caregiving hours for children, and time of care for children with SCA were 6±2.5, 5±4.7 hours, and 8±4.2 years, respectively (Table 1). Among the families, 58 (46.8%) had more than one family member diagnosed with SCA, and 74 (59.7%) had a family member diagnosed with the SCA trait. Additionally, 86 (69.4%) caregivers were not diagnosed with associated physical or psychological diseases. Furthermore, 58 (46.8%) of families provided healthcare to more than one person in the family, and 110 (88.7%) reported having assistance in caring for children (Table 2).

**Table 1:** Sociodemographic characteristics of caregivers of children with SCA: categorical and continuous variables *SAR: Saudi Arabia riyal; SCA: sickle cell anemia; SD: standard deviation.

Characteristic	Category	n (%)
Caregiver relationship with the patient	Father	83 (66.9)
	Mother	41 (33.1)
Caregivers’ age	≤40 years	52 (41.9)
	>40 years	72 (58.1)
Nationality	Saudi	96 (77.4)
	Non-Saudi	28 (22.6)
Marital status	Married	115 (92.7)
	Divorced	9 (7.3)
Caregivers’ educational level	Primary school	22 (17.7)
	Intermediate or high school	50 (40.3)
	University or higher	52 (41.9)
Caregivers’ job status	Government sector employee	36 (29)
	Private sector employee	25 (20)
	Not employed	63 (50.8)
Caregivers’ income (SAR)*	<5000	62 (50)
	5000–9999	25 (20.2)
	>10,000	37 (29.8)
	Mean (SD)	Minimum–Maximum
Number of family members	6 (2.5)	1–18
Average caregiving for children (years)	8 (4.2)	0.8–14
Average daily caregiving hours for children	5 (4.7)	0.0–13

**Table 2:** Sociodemographic characteristics of children with SCA and their families: categorical variables SCA: sickle cell anemia.

Characteristic	Response	n (%)
Had any family member diagnosed with SCA?	Yes	58 (46.8)
	No	66 (53.2)
Had any family member diagnosed with SCA trait?	Yes	74 (59.7)
	No	50 (40.3)
Caregiver diagnosed with physical or psychological diseases	No	86 (69.4)
	Yes	38 (30.6)
Provided healthcare to more than one person in the family	Yes	58 (46.8)
	No	66 (53.2)
Had any assistance in caring for children	Yes	110 (88.7)
	No	14 (11.3)
Children’s sex	Boy	67 (54.0)
	Girl	57 (46.0)
Children’s age	≤9 years	67 (54.0)
	>9 years	57 (46.0)
Had a child who received hydroxyurea treatment?	Yes	74 (59.7)
	No	50 (40.3)
Had an affected child diagnosed with physical or psychological diseases?	No	94 (75.8)
	Yes	30 (24.2)

Overall, 67 (54.0%) of children were boys, and 67 (54.0%) were aged ≤9 years. Regarding treatment, 74 (59.7%) of children received hydroxyurea, whereas 50 (40.3%) did not. Among the children, 30 (24.2%) were diagnosed with associated physical or psychological diseases (Table 2).

The analysis of the ZBI scores revealed that 45 (36.3%) of caregivers reported no burden, whereas 51 (41.1%), 22 (17.7%), and 6 (4.8%) reported mild, moderate, and severe burden, respectively (Table 3). Regarding factors associated with caregiver burden for children with SCA, mothers were more likely to experience burden than fathers (odds ratio [OR]: 3.8, 95% CI: 1.58-9.10). Non-Saudi caregivers exhibited a higher likelihood of burden than Saudi caregivers (OR: 3.0, 95% CI: 1.20-7.56). Unemployed caregivers were more likely to experience burden than those employed in the government sector (OR: 5.1, 95% CI: 1.40-18.69). Additionally, caregivers with an income of <5000 SAR had a higher likelihood of burden than those with an income of >10000 SAR (OR: 10.3, 95% CI: 2.27-46.96) (Table 4). Mean caregiving hours per day were higher among caregivers who reported burden (8.7±4.6) compared with those among caregivers who did not (3.6±4.1; p<0.001) (Table 5). Caregivers of children diagnosed with physical or psychological diseases had a higher likelihood of experiencing burden than their counterparts (OR: 4.0, 95% CI: 1.62-9.99) (Table 6).

**Table 3:** SCA caregiver burden SCA: sickle cell anemia.

Zarit Burden Interview	
Burden level	n (%)
No burden	45 (36.3)
Mild	51 (41.1)
Moderate	22 (17.7)
Severe	6 (4.8)

**Table 4:** Association between caregiver burden and sociodemographic characteristics of caregivers SAR: Saudi Arabia riyal; OR: odds ratio; CI: confidence interval; P<0.05, significant values.

Characteristic		Burden		OR	95% CI	P-value
		Yes (%)	No (%)			
Caregiver relationship with the patient	Mother	16 (39)	25 (61.0)	3.8	(1.58–9.10)	0.002
	Father	12 (14.5)	71 (85.5)			
Caregivers’ age	≤40 years	16 (30.8)	36 (69.2)	2.2	(0.95–5.23)	0.064
	>40 years	12 (16.7)	60 (83.3)			
Nationality	Non-Saudi	11 (39.3)	17 (60.7)	3.0	(1.20–7.56)	0.016
	Saudi	17 (17.7)	79 (82.3)			
Marital status	Married	27 (23.5)	88 (76.5)	2.5	(0.29–20.51)	0.393
	Divorced	1 (11.1)	8 (88.9)			
Caregivers’ educational level	Primary school	2 (9.1)	20 (90.9)	0.3	(0.07–1.64)	0.176
	Intermediate or high school	14 (28)	36 (72)	1.3	(0.53–3.17)	0.569
	University or higher (Ref.)	12 (23.1)	40 (76.9)	1		
Caregivers’ job status	Government sector employee	3 (8.3)	33 (91.7)	1		
	Private sector employee	5 (20)	20 (80)	2.8	(0.53–12.77)	0.197
	Not employed	20 (31.7)	43 (68.3)	5.1	(1.40–18.69)	0.014
Caregivers’ income (SAR)	<5000	23 (37.1)	39 (62.9)	10.3	(2.27–46.96)	0.003
	5000–9999	3 (12)	22 (88)	2.4	(0.37–15.44)	0.361
	>10,000 (Ref.)	2 (5.4)	35 (94.6)	1		

**Table 5:** Association between caregiver burden and family characteristics SD: standard deviation; mean diff.: mean difference; CI: confidence interval; P<0.05 significant values.

Characteristic	Burden		Mean diff. (95% CI)	P-value
	Yes	No		
Number of family members, mean (SD)	5.7 (2.6)	6.0 (2.4)	−0.32 (−1.4 to 0.8)	0.270
Average caregiving for children (years), mean (SD)	8.3 (4.5)	7.8 (4.2)	4.7 (−1.3 to 2.3)	0.305
Average daily caregiving hours for children, mean (SD)	8.7 (4.6)	3.6 (4.1)	5.1 (5.1–7.0)	<0.001

**Table 6:** Association between caregiver burden and sociodemographic characteristics of children with SCA and their families SCA: sickle cell anemia; OR: odds ratio; CI: confidence interval; P<0.05 significant values.

Characteristic		Burden		OR	95% CI	P-value
		Yes (%)	No (%)			
Had any family member diagnosed with SCA?	Yes	17 (29.3)	42 (70.7)	2.1	(0.88–4.90)	0.093
	No	11 (16.7)	55 (83.3)			
Had any family member been diagnosed with the SCA trait?	Yes	20 (27)	54 (73)	1.9	(0.78–4.85)	0.150
	No	8 (22.6)	42 (84.0)			
Had a child in the family who had physical or psychological diseases?	Yes	10 (26.3)	28 (73.7)	1.4	(0.55–3.28)	0.508
	No	18 (20.9)	68 (79.1)			
Provided healthcare to more than one person in the family?	Yes	17 (29.3)	41 (70.7)	2.1	(0.87–4.90)	0.093
	No	11 (16.7)	55 (83.3)			
Had any assistance in caring for children?	Yes	24 (21.8)	86 (78.2)	0.7	(0.20–2.42)	0.517
	No	4 (28.6)	10 (71.4)			
Sex	Male	15 (22.4)	52 (77.6)	0.97	(0.42–2.27)	0.956
	Female	13 (22.8)	44 (77.2)			
Age	≤9	16 (23.9)	51 (76.1)	1.2	(0.50–2.75)	0.707
	>9	12 (21.1)	45 (78.9)			
Had a child who received hydroxyurea treatment?	Yes	18 (24.3)	56 (75.7)	1.3	(0.54–3.08)	0.572
	No	10 (20)	40 (80)			
Had a child diagnosed with physical or psychological diseases?	Yes	13 (43.3)	17 (56.7)	4.0	(1.62–9.99)	0.002
	No	15 (16)	79 (84)			

Multiple logistic regression analysis revealed that caregivers of children diagnosed with physical or psychological diseases had a higher likelihood of experiencing burden than caregivers of children without such diagnoses (OR: 6.9, 95% CI: 2.0-23.8, p = 0.001). Additionally, caregivers with a monthly income of <5000 SAR were more likely to experience a burden than those with an income >10,000 SAR (OR: 10.9, 95% CI: 1.8-64.8, p = 0.009). These findings indicated 20% higher odds of experiencing burden for every one-hour increase in caregiving duration (OR: 1.2, 95% CI: 1.1-1.4, p<0.001) (Table 7).

**Table 7:** Factors associated with burden on caregivers of children with SCA: multiple logistic regression analysis SCA: sickle cell anemia; SAR: Saudi Arabia riyal; OR: odds ratio; CI: confidence interval; P<0.05 significant values.

Characteristic	Response	B	Wald	P-value	OR	95% CI
Had a child diagnosed with physical or psychological diseases?	Yes	−1.92	9.2	0.001	6.9	2.0–23.8
	No				1	
Monthly income	<5000 SAR	2.39	6.9	0.009	10.9	1.8–64.8
	5000–9999 SAR	0.14	0.02	0.898	1.2	0.1–9.8
	>10,000 (Ref.)				1	
Average daily caregiving hours for children		0.20	13.3	<0.001	1.2	1.1–1.4

## Discussion

Our study sheds light on the burden faced by family caregivers of children with SCA, identifying key demographic and socioeconomic factors contributing to this burden. The results show that the average number of family members, the percentage of caregivers being unemployed, and those with a low income of <5000 SAR were 6±2.5, 50.8%, and 50%, respectively. Furthermore, mothers had a higher likelihood of experiencing a burden than fathers.

Further insights from the study also examined sex distribution, age groups, and the presence of coexisting physical or psychological diseases in children with SCA and found a higher prevalence of SCA in boys (54.0%) and a higher proportion of affected children aged ≤ 9 years (54.0%). Additionally, our results reveal that caregivers of children diagnosed with physical or psychological diseases had a higher likelihood of experiencing burden, highlighting additional challenges faced by caregivers in managing younger children with coexisting health conditions.

Madani et al. reported similar characteristics among caregivers of children with SCA in western Saudi Arabia, showing that larger families (average of 5±2.0 members) with limited financial resources (64.5%), housewife mothers (79.4%), and those lacking social assistance or health insurance (80%) experienced significant financial and emotional burdens [8]. These burdens affected various aspects of their quality of life, including their social and professional lives [8].

Kuerten et al. aimed to characterize the psychosocial burden experienced by caregivers of children with SCA in Kenya and identified predictors of the psychosocial burden, including disease severity and financial hardship. Their findings align with our results, suggesting that caregivers of children with SCA experienced difficulties across multiple domains of functioning and that financial hardships were likely to be associated with psychosocial burdens [5].

Regarding caregiving hours, the study data showed that caregivers who devoted more hours to caregiving responsibilities were more likely to experience burden, suggesting that caregiving duration contributes to the overall burden experienced by caregivers. The result of this study is consistent with that of the existing literature, including a study by Madani et al. who examined the quality of life among caregivers of patients with SCA and highlighted that caregivers expressed the lowest satisfaction levels in certain areas, such as financial situation, free time activity, and life environment [8]. Healthcare providers and support organizations should develop comprehensive strategies to alleviate the burden and promote overall well-being among caregivers by considering financial resources, caregiving hours, and the presence of coexisting health conditions. Our findings indicate that 36.3% of caregivers reported no burden, whereas 41.1%, 17.7%, and 4.8% had mild, moderate, and severe burden, respectively, based on the ZBI score. The reported caregiver burden varies among studies that used the ZBI. For example, a study conducted by Grygiel et al. in Grenada found that 53.8% of caregivers reported little to no burden, whereas 34.6% and 11.5% reported mild and moderate burden, respectively, with no reported cases of severe burden [11]. By contrast, another study by Baker et al. in Jamaica reported that 26% of caregivers experienced little to no burden, whereas 61%, 12%, and 1% reported mild, moderate, and severe burden, respectively [10].

The strength of this cross-sectional study lies in selecting the Maternity and Child Hospital at King Salman Medical City in Madinah, the primary governmental hospital in the region, which provides comprehensive healthcare services and specialized care. This choice enables extensive data collection from all registered files of children receiving treatment for SCA in the Madinah region, along with a robust response rate of 74.7% from family caregivers. This approach offers valuable insights into caregiver burden and the influence of sociodemographic and clinical factors in contributing to caregiving burden.

Nevertheless, this study has some limitations, which must be considered when interpreting the findings. The cross-sectional nature of this study only provided a snapshot of caregiver burden, which may vary over time and in different disease situations. Furthermore, the single-center design restricts the applicability of our findings to a broader population. Future longitudinal studies with larger and more diverse samples should be conducted to address these limitations and further validate and generalize the findings.

## Conclusions

This study highlights the importance of understanding the intricate dynamics within families affected by SCA and the sociodemographic characteristics contributing to caregiver burden. Our results indicate that caregivers of SCA children with physical or psychological diseases were more susceptible to experiencing burden, particularly regarding caregiving hours. Furthermore, caregivers with lower incomes were more likely to experience a burden. Targeted interventions and comprehensive support programs can be developed by acknowledging the presence of vulnerable populations and identifying the factors associated with caregiver burden to alleviate the burden and enhance the overall well-being of family caregivers.

## Appendices

**Table 8:** Questionnaire for measuring the caregiver burden of children with sickle cell anemia Interpretation of Score: total score as follows: 0–20, little or no burden; 21–40, mild to moderate burden; 41–60, moderate to severe burden; and 61–88, severe burden

First part: sociodemographic data of the caregivers							
What is your relationship with the patient?	- Fathers						
	- Mothers						
	- Grandfathers and grandmothers						
	- Brothers and sisters						
	- Uncles, Aunts						
Phone number							
Age							
Gender	Male				Female		
Nationality	Saudi				Non-Saudi		
Marital status	Married			Widowed		Divorced	
Number of family members							
Is there anyone from the same family (brother and sisters) who has sickle cell anemia?	YES				NO		
Is there anyone from the same family (brother and sisters) who has sickle cell anemia trait?	YES				NO		
Does anyone from the same family (brother and sister of the affected child) have a physical or psychological disease?	NO				- Diabetes mellitus		
					- Hypertension		
					- Heart disease		
					- Kidney disease		
					- Liver disease		
					- Cancer (Breast, colon and rectum...)		
					- Depression disorder		
					- Anxiety disorder		
					- Bipolar disorder		
					- Schizophrenia disorder		
					- Post-traumatic stress disorder		
					-Others (…………..)		
Do you provide health care to more than one person in the same family?	YES				NO		
How long do you care for your child measured in years?	- Less than 1						
	-1						
	-2						
	-3						
	-4						
	-5						
	-6						
	-7						
	-8						
	-9						
	-10						
	-11						
	-12						
	-13						
	-14						
Is there someone outside the hospital who helps you in providing care for the same patient?	YES				NO		
Who is the person helping you with providing care for the sickle cell anemia child?	-Father						
	-Mother						
	-Grandfather or Grandmother						
	-Brothers or Sisters						
	-Uncles and Aunts						
How much time is typically spent on caregiving of the sickle cell anemia child on a daily basis, on average measured in hours?	- Less than 1						
	- 1						
	- 2						
	- 3						
	- 4						
	- 5						
	- 6						
	- 7						
	- 8						
	- 9						
	- 10						
	- 11						
	- 12						
	- More than 12						
Educational level	- Inability to read or write						
	- Primary						
	- Elementary						
	- Secondary						
	- Bachelor's degree and more						
Job Status	-Government sector employee						
	-Private sector employee						
	-Not employed						
	-Student						
	-Housewife						
	-Have your own business						
	-Retired						
Monthly income	-Less than 5000 SR						
	-5000-9999 SR						
	-10000-15000 SR						
	-More than 15000 SR						
Reason for no work	- Has a relationship to your care of the patient						
	- Has not related to your care of the patient						
Second Part: sociodemographic data of the child							
Gender	Male				Female		
Age measured in years ?							
How long since diagnosis with sickle cell anemia measured in years ?	- Less than 1						
	- 1						
	- 2						
	- 3						
	- 4						
	- 5						
	- 6						
	- 7						
	- 8						
	- 9						
	- 10						
	- 11						
	- 12						
	- 13						
	- 14						
Is the child receive Hydroxyurea treatment ?	YES				NO		
Has the child been diagnosed with other diseases, whether physical or psychological?	NO				- Diabetes mellitus		
					- Developmental disorders		
					- Liver disease		
					- kidney disease		
					- Heart disease		
					- Autism Spectrum Disorders		
					- Attention-Deficit/Hyperactivity Disorder (ADHD)		
					- Anxiety disorder		
					- Others (……………)		
Third Part: Zarit Burden Interview							
Questions Please circle the response the best describes how you feel.	Score						
	Never 0	Rarely 1	Some-times 2		Quite- frequently 3		Nearly always 4
1 Do you feel that your relative asks for more help than he/she needs?	0	1	2		3		4
2 Do you feel that because of the time you spend with your relative that you don’t have enough time for yourself?	0	1	2		3		4
3 Do you feel stressed between caring for your relative and trying to meet other responsibilities for your family or work?	0	1	2		3		4
4 Do you feel embarrassed over your relative’s behaviour?	0	1	2		3		4
5 Do you feel angry when you are around your relative?	0	1	2		3		4
6 Do you feel that your relative currently affects our relationships with other family members or friends in a negative way?	0	1	2		3		4
7 Are you afraid what the future holds for your relative?	0	1	2		3		
8 Do you feel your relative is dependent on you?	0	1	2		3		4
9 Do you feel strained when you are around your relative?	0	1	2				4
10 Do you feel your health has suffered because of your involvement with your relative?	0	1	2		3		4
11 Do you feel that you don’t have as much privacy as you would like because of your relative?	0	1	2		3		4
12 Do you feel that your social life has suffered because you are caring for your relative?	0	1	2		3		4
13 Do you feel uncomfortable about having friends over because of your relative?	0	1	2		3		4
14 Do you feel that your relative seems to expect you to take care of him/her as if you were the only one he/she could depend on?	0	1	2		3		4
15 Do you feel that you don’t have enough money to take care of your relative in addition to the rest of your expenses?	0	1	2		3		4
16 Do you feel that you will be unable to take care of your relative much longer?	0	1	2		3		4
17 Do you feel you have lost control of your life since your relative’s illness?	0	1	2		3		4
18 Do you wish you could leave the care of your relative to someone else?	0	1	2		3		4
19 Do you feel uncertain about what to do about your relative?	0	1	2		3		4
20 Do you feel you should be doing more for your relative?	0	1	2		3		4
21 Do you feel you could do a better job in caring for your relative?	0	1	2		3		4
22 Overall, how burdened do you feel in caring for your relative?	0	1	2		3		4
Total							
